# Pannus formation: a rare culprit of early bioprosthetic valve dysfunction—a case report

**DOI:** 10.1093/ehjcr/ytae518

**Published:** 2024-10-03

**Authors:** Sho Takemoto, Hiroshi Kumano, Junichi Shimamura, Akira Shiose

**Affiliations:** Center for Transplantation Sciences, Department of Surgery, Massachusetts General Hospital/Harvard Medical School, 13th Street, Building 149, Boston, MA 02129, USA; Department of Cardiovascular Surgery, Kyushu University Hospital, 3-1-1 Maidashi, Higashi-ku, Fukuoka 812-8582, Japan; Department of Cardiovascular Surgery, Northern Okinawa Medical Center, 1712-3, Umusa, Nago, Okinawa 905-8611, Japan; Division of Cardiothoracic Surgery, Westchester Medical Center, 100 Woods Rd, Valhalla, NY 10595, USA; Department of Cardiovascular Surgery, Kyushu University Hospital, 3-1-1 Maidashi, Higashi-ku, Fukuoka 812-8582, Japan

**Keywords:** Pannus, Bioprosthetic valve dysfunction, Surgical aortic valve replacement, Elderly patient, Case report

## Abstract

**Background:**

Early bioprosthetic valve dysfunction (BVD) due to pannus formation is uncommon in elderly patients, and only a limited number of cases have been reported.

**Case summary:**

An 84-year-old man presented with exertional dyspnoea 3 years after surgical aortic valve replacement (SAVR) with a 19 mm Epic™ valve (Abbott, Santa Clara, CA, USA). Transthoracic echocardiography demonstrated progressive BVD, and cardiac computed tomography (CT) revealed sub-aortic pannus formation. Re-operative SAVR was performed using a 19 mm INSPIRIS RESILIA® valve (Edwards Lifesciences, Irvine, CA, USA), and pathological examination confirmed valve leaflet deformation caused by pannus overgrowth. At the 18-month follow-up, the patient exhibited favourable progress, with no indications of BVD or pannus recurrence.

**Discussion:**

This case highlights the importance of recognizing early pannus formation as a cause of BVD, even in elderly patients. Early detection of BVD based on clinical symptoms and echocardiography is vital to allow timely surgical intervention before the deterioration of cardiac function. Cardiac CT helps to differentiate pannus from thrombus formation and guide treatment decisions.

**Learning points**

- Pannus overgrowth should be considered a rare but important cause of early bioprosthetic valve dysfunction, even in elderly patients.
- Contrast-enhanced cardiac computed tomography is helpful in differentiating pannus from thrombus formation.

## Introduction

Pannus formation in prosthetic valves commonly occurs ∼5 years after implantation, leading to bioprosthetic valve dysfunction (BVD).^1^ The incidence of pannus formation is comparable between bioprosthetic and mechanical valves,^1^ with a 10-year cumulative incidence of 0.3%.^2^ The development of pannus is linked to inflammatory reactions, but the exact aetiology remains unclear, and a limited number of early BVD within 5 years have been reported.^3^ Herein, we present a case of an 84-year-old man who underwent re-operative surgical aortic valve replacement (SAVR) for BVD caused by severe pannus formation only 3 years after the initial operation.

## Summary figure

**Figure:**
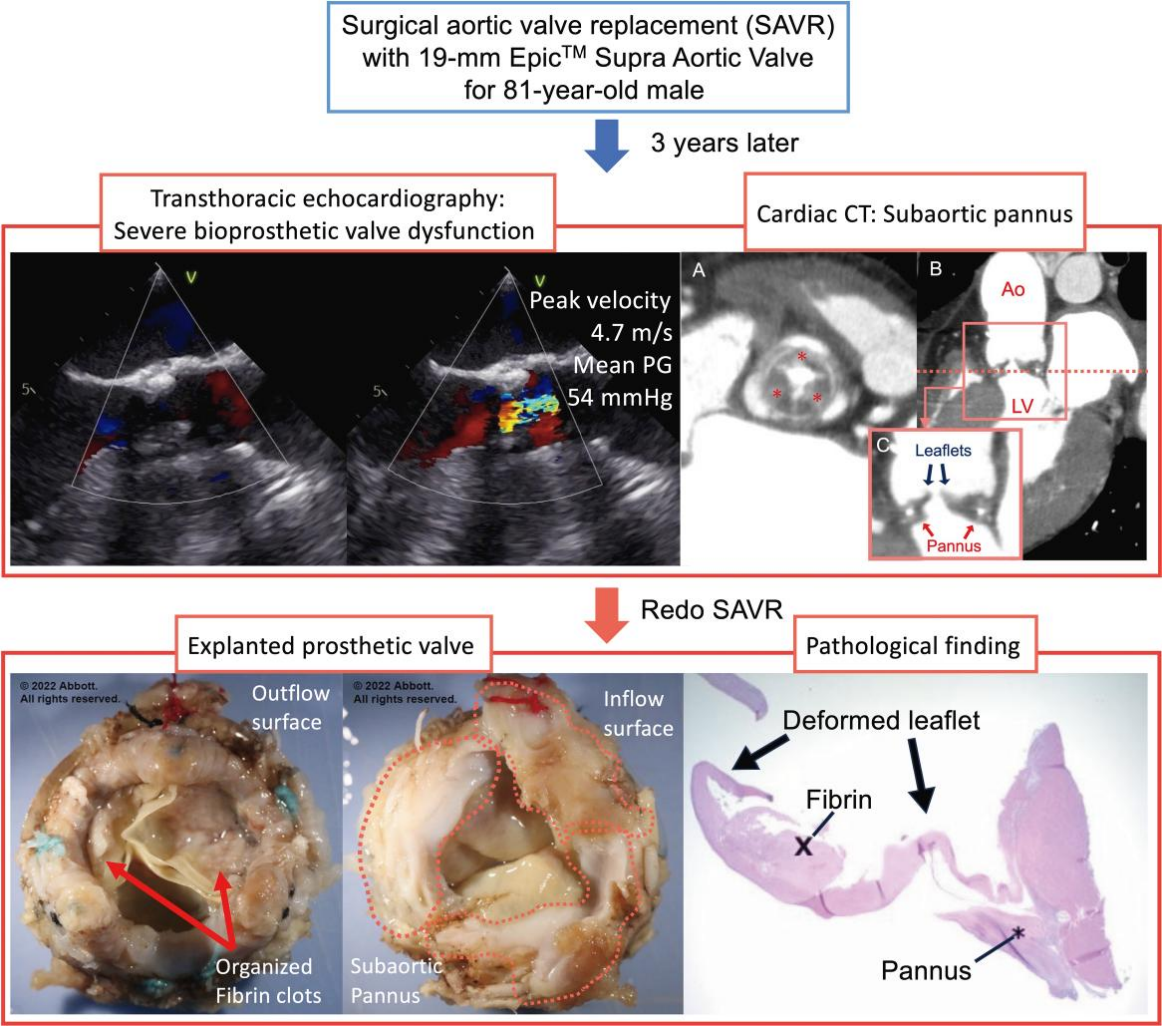


## Case presentation

**Figure 1:**
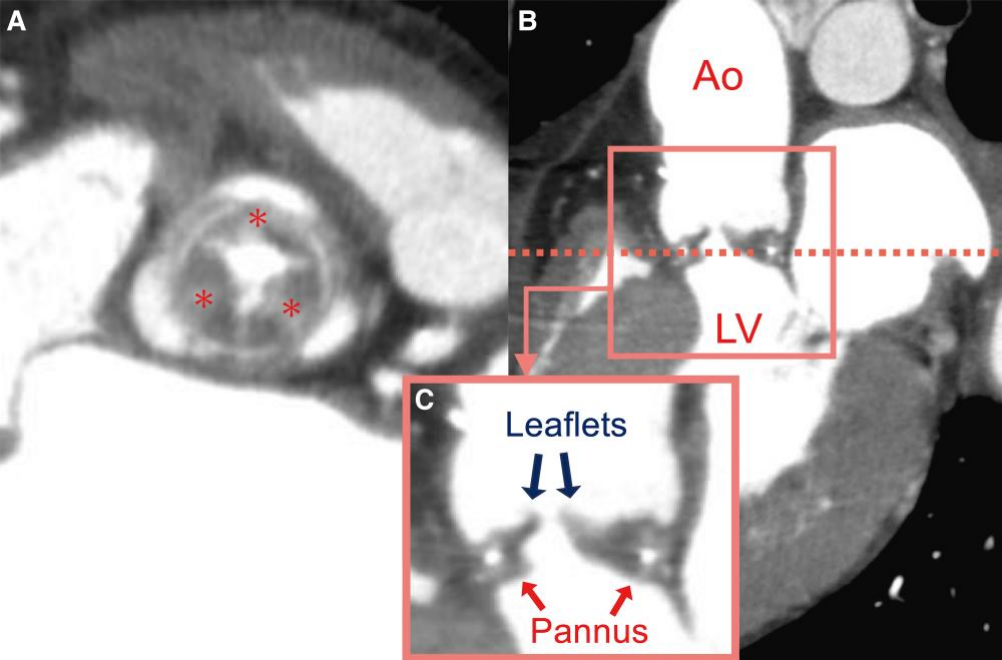
*(A)* Short axis view of the bioprosthetic valve in contrast-enhanced computed tomography. This image is a cross-section at the level of the dotted line in *(B*). Asterisks: pannus beneath all three valve leaflets. (*B*) Sagittal view of the prosthetic valve (Ao, aorta; LV, left ventricle). (*C*) Enlarged view of the area enclosed by the square in *(B)*.

An 84-year-old man presented with progressive exertional dyspnoea 3 years after undergoing SAVR with a 19 mm Epic™ Supra Aortic Valve (Abbott, Santa Clara, CA, USA) through a median sternotomy for severe aortic stenosis. The patient had a history of hypertension and hyperuricaemia, which were monitored without medication. Pre-operative echocardiography measured the aortic annulus at 19 mm. During the initial operation, the 21 mm sizer did not pass through the annulus. Therefore, a 19 mm valve was implanted. His body surface area (BSA) was 1.52 m^2^, and the estimated effective orifice area index was 0.97 cm^2^/m^2^. Transthoracic echocardiography (TTE) performed on the 11th post-operative day revealed a high-flow status with a trans-prosthetic valvular peak velocity of 3.0 m/s and a mean pressure gradient (PG) of 20.8 mmHg. Two years post-SAVR, TTE indicated progressive BVD with a peak velocity of 4.7 m/s and a mean PG of 54 mmHg; however, no paravalvular leakage was observed. Medical optimization was attempted including oral frusemide 40 mg daily and oral spironolactone 50 mg daily, but the patient developed progressive exertional dyspnoea, requiring readmission for further management 3 years after the initial operation. A physical examination revealed a harsh systolic murmur at the right upper sternal border, bilateral lung crackles, and mild bilateral leg oedema. His body weight decreased from 50 kg to the low 40 kg range over the 3 years following the initial operation, due to improved dietary habits, ageing, and muscle loss. At the time of readmission, his body weight was 46 kg, a 3 kg increase from his recent baseline, and his BSA was 1.41 m². His heart rate was 124 b.p.m., blood pressure measured 134/98 mmHg, and oxygen saturation level was 93% on 2 L of inhaled oxygen. The patient was classified as a New York Heart Association (NYHA) functional class III. A chest radiograph revealed bilateral pulmonary congestion. Blood tests showed no anaemia, normal kidney function, and a significantly elevated brain natriuretic peptide level of 1236 pg/mL. In addition, the TTE revealed a decreased left ventricular ejection fraction, declining from 63 to 45% compared with the previous year. Contrast-enhanced computed tomography (CT) revealed hypertrophic tissue overhanging the aortic valve, suggesting pannus formation (*Figure 1*), and a re-operative SAVR was planned.

**Figure 2:**
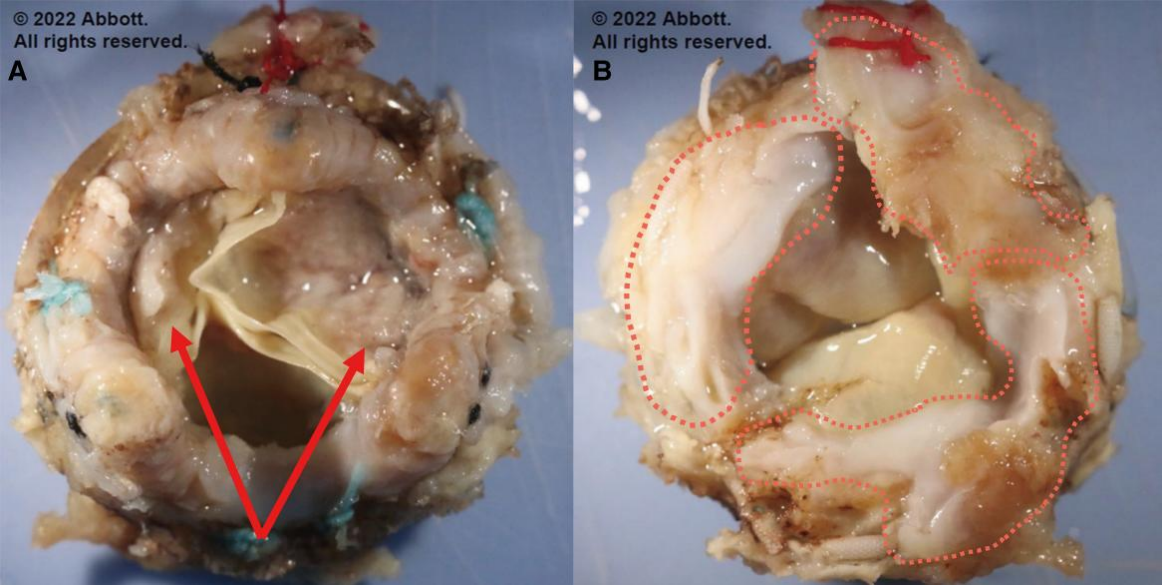
*(A)* The outflow surface of the removed bioprosthetic valve. Arrowheads: organized fibrin clots on the leaflets. (*B*) The inflow surface of the removed bioprosthetic valve. The area enclosed by dotted lines indicates pannus formation and growth over the valve leaflets.

**Figure 3:**
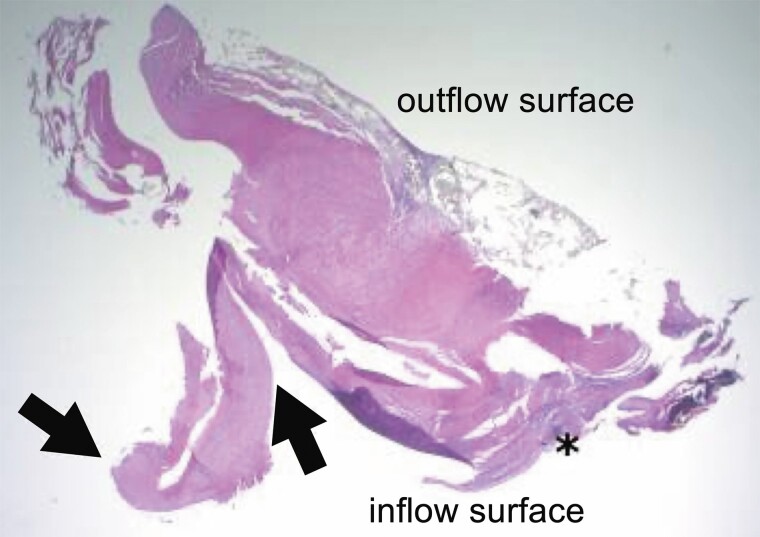
Pathological findings of the valve leaflet. Asterisk: pannus formation. Arrowheads: the valve leaflet is captured and bent by the pannus.

Following resternotomy, a cardiopulmonary bypass (CPB) was established, and cardiac arrest was induced. The prosthetic valve was inspected via re-aortotomy, and the surfaces of all 3 valve leaflets were covered with organized fibrin clots. Subsequent removal of the prosthetic valve revealed pannus formation and growth over the valve leaflets on the inflow surface, which restricted leaflet mobility (*Figure 2*). Pathological examination of the prosthetic valve revealed that the pannus had captured the valve leaflet and caused it to bend midway (*Figure 3*). The pannus consisted of fibrous tissue with chronic inflammation and calcification; no acute inflammation was observed. A 19 mm INSPIRIS RESILIA® valve (Edwards Lifesciences, Irvine, CA, USA) was implanted supra-annularly. The patient was subsequently weaned off CPB with intra-aortic balloon pump support. The post-operative course was complicated by a complete atrioventricular block that required the implantation of a permanent pacemaker. The patient was eventually discharged home following the stay at a rehabilitation hospital. At the 18-month post-operative follow-up, his condition improved from an NYHA class III pre-operatively to class I post-operatively; moreover, TTE revealed excellent valve function with a peak velocity of 2.3 m/s and a mean PG of 9 mmHg. No signs of pannus formation were observed.

## Discussion

Sub-aortic pannus formation at the prosthetic valve is infrequent, with reported cumulative incidences of 0.3, 5.0, and 9.9% at 10, 20, and 25 years after implantation, respectively.^2^ Young age, smaller prosthetic valve size, and concurrent mitral valve replacement have been identified as risk factors for pannus formation.^2^ The differential diagnosis of pannus formation includes thrombus formation; anticoagulation therapy is an option in patients with thrombi responsible for BVD. However, surgical management is recommended in patients with severe BVD secondary to pannus formation. The guideline of the European Society of Cardiology and the European Association for Cardio-Thoracic Surgery emphasizes the significance of distinguishing these two aetiologies by cardiac CT.^4^ Pannus formation is characterized by a higher radiological density than that of thrombi (Hounsfield units > 145 and > 90, respectively).^1^ In our case, cardiac CT guided the optimal treatment, highlighting the importance of correctly identifying the underlying cause of BVD. However, definitive long-term management to prevent pannus recurrence remains to be established. Close follow-up with serial echocardiography is essential, and cardiac CT could be considered if BVD is suspected based on echocardiography. Routine cardiac CT scans, especially in elderly patients, offer limited benefits due to the associated risk of kidney impairment. Nevertheless, early detection allows timely surgical intervention before heart failure worsens, ensuring better outcomes.

As of March 2024, three cases of pannus-induced early Epic™ valve dysfunction within 5 years post-implantation have been reported.^5–7^ Higher immunologic activity in younger individuals is considered to accelerate earlier valve degeneration and pannus formation.^3^ The ages of the patients in previous reports ranged from 46 to 75 years, whereas the patient in our case was in his 80s.^5–7^ Guenzinger *et al*.^8^ analysed 455 patients who underwent SAVR with the Biocor™ valve, the previous model before the Epic™, and reported a 10-year freedom from valve-related reoperation of 90.6 ± 1.7%. None of the patients who required valve-related reoperations exhibited pannus-induced valve dysfunction over the follow-up period of 8.4 ± 5.6 years. The mechanism of early pannus formation specific to the Epic™ valve was unclear, but we opted to utilize a different prosthetic valve to mitigate the risk of early pannus recurrence. In the Commence trial, which comprised a patient cohort with a mean age of 65.1 ± 10.9 years, the INSPIRIS RESILIA® valve demonstrated a 5-year freedom from structural valve deterioration of 100%, and a 7-year freedom of 99.3%.^9^ These excellent results prompted us to choose this valve for our patient.

A recent meta-analysis demonstrated that aortic annular enlargement (AAE) does not increase surgical risk during the initial operation.^10^ While 19 mm valves showed comparable outcomes with 21 mm valves in patients with small BSA,^11^ a larger valve with AAE could have potentially prevented early pannus formation, as smaller prosthetic valves can be a risk. During the reoperation, concerns arose regarding early pannus recurrence with another 19-mm valve. However, we did not perform AAE because of surgical risks such as patient age, re-operative surgery, worsening cardiac function, and the potential risk of prolonging aortic cross-clamping and CPB time.

Valve-in-valve transcatheter aortic valve replacement (ViV-TAVR) was deemed unsuitable due to concerns about inadequate valve expansion in a valve with pannus overgrowth, which has been associated with hypo-attenuating leaflet thickening and may lead to early BVD.^12^ Furthermore, if BVD recurred after ViV-TAVR, another ViV-TAVR would present significant risks of valve expansion failure and coronary artery obstruction. In such a scenario, a higher-risk TAVR explant would be the only option. These considerations led us to choose redo SAVR rather than ViV-TAVR even in an elderly patient. Early surgical intervention before the deterioration of cardiac function should be considered once BVD is diagnosed.

## Conclusions

In conclusion, we have presented the case of a patient with early BVD who required reoperation for pannus formation. Physicians should acknowledge that pannus formation is a significant cause of early BVD, although it is rare in elderly patients.

## Lead author biography


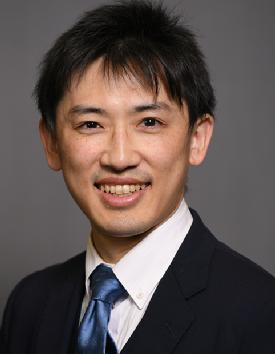


Sho Takemoto is a cardiac surgeon who completed his cardiovascular surgery training in Japan. Currently, he is a research fellow at the Center for Transplantation Sciences, Massachusetts General Hospital, where he focuses on advancing the fields of allo- and xeno-transplantation and *ex vivo* heart perfusion.

## Data Availability

The data underlying this article will be shared on reasonable request to the corresponding author.
